# The Role of Lipid Metabolism in the Progression of Breast Cancer

**DOI:** 10.7150/jca.128498

**Published:** 2026-04-08

**Authors:** Tong-Tong Liu, Meng-Meng Wang

**Affiliations:** 1 Department of Pharmacy, Fudan University Shanghai Cancer Center, Shanghai, China.; 2 Department of Oncology, Shanghai Medical College of Fudan University, Shanghai, China.

**Keywords:** lipid metabolism, breast cancer, fatty acid synthase, cluster of differentiation 36, acyl-CoA synthetase long chain family member 4

## Abstract

Breast cancer is one of the most prevalent malignancies globally, it is closely associated with lipid metabolism reprogramming. Targeting lipid metabolism pathways has become a promising strategy for breast cancer treatment. Lipid metabolism encompasses the biochemical processes of lipid biosynthesis, catabolism, uptake, and post-synthetic modification. Dysregulation of these processes can promote tumorigenesis and cancer cell metastasis. This review summarizes four major facets of lipid metabolism, including fatty acid (FA) metabolism, cholesterol metabolism, phospholipid (PL) metabolism, and sphingolipid metabolism. It also discusses the roles of key molecules in these pathways in breast cancer, including FA synthase (FASN), cluster of differentiation 36 (CD36), and acyl-CoA synthetase long-chain family member 4 (ACSL4). These molecules may improve therapeutic responses, overcome drug resistance, and reshape the tumor immune microenvironment by regulating FA synthesis and lipid uptake. Targeting lipid metabolic pathways may provide potential biomarkers for patient stratification and therapeutic guidance, and may also offer new opportunities for cancer treatment.

## Introduction

Breast cancer is the most common malignancy worldwide and the primary cause of female cancer mortality 1. Recent years have witnessed a slight decrease in breast cancer mortality rates, driven primarily by increased public awareness, earlier diagnosis, more cost-effective screening programs, and improved treatment technologies 2. However, the heterogeneity of breast cancer makes it a profoundly challenging solid tumor, constituting a major diagnostic and therapeutic hurdle.

Lipids are an important energy source and are composed of three structural domains: a polar head group, a hydrophobic tail, and a connecting linker 3. The primary classes of lipids are fatty acids (FAs), cholesterol, phospholipids (PL), and sphingolipids. Lipids play the role of key signaling molecules in tumors, and elevated lipid metabolism is a hallmark of malignant cancers. Cancer cells meet their lipid demands by coordinating lipid uptake, synthesis, transport, storage, and catabolism. Studies have shown that even when exogenous lipids are abundant in the tumor microenvironment, cancer cells continue to abnormally activate de novo lipid synthesis, a phenomenon regarded as a hallmark of metabolic reprogramming 4. Lipid metabolism is often drastically altered at different stages of tumor development. The main mechanisms include the regulation of ferroptosis, the provision of energy for tumor metastasis, and metabolic reprogramming of cells within the tumor microenvironment, all of which promote tumor growth and survival 5. This review summarizes current research on the role of lipid metabolism in breast cancer pathogenesis, elucidates the underlying molecular signaling pathways and mechanistic drivers linking dysregulated lipid metabolism to tumor development and progression, and proposes targeted modulation of lipid metabolic pathways as a promising therapeutic and preventive strategy for breast cancer.

## 1. Lipid metabolism

**Figure 1:**
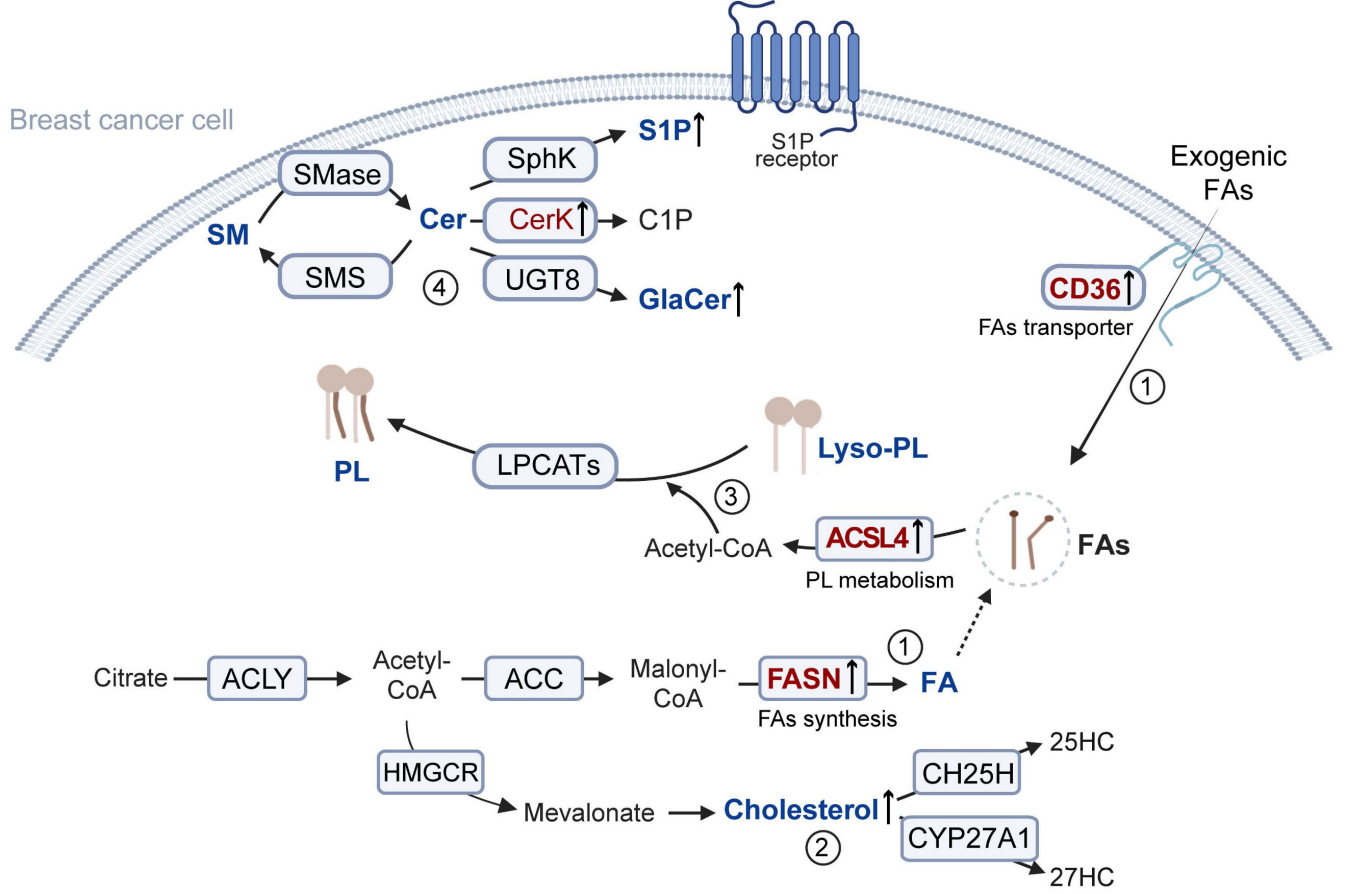
Overview of lipid metabolism. **1.** Citrate derived from mitochondria is cleaved by ACLY to generate cytosolic acetyl-CoA. This acetyl-CoA is carboxylated to malonyl-CoA and subsequently elongated into FAs under the high expression of FASN, supporting the rapid proliferation of tumor cells. Moreover, tumor cells can enhance the uptake of exogenous FAs via CD36-mediated pathways. **2.** Acetyl-CoA is also utilized in cholesterol biosynthesis, thereby leading to the accumulation of cholesterol. **3.** The increased FAs are further incorporated into phospholipid metabolism. Specifically, FAs are converted into acyl-CoA by ACSLs, and subsequently attached to the sn-2 position of Lyso-PLs by LPCAT3, producing phospholipids involved in membrane remodeling and signaling. **4.** The primary metabolic product of sphingomyelin is Cer. It can be reconverted into sphingolipids by sphingolipid synthases. Cer can also promote the catalytic activity of various kinases, generating multiple bioactive derivatives, including S1P, C1P, and GalCer. SM: Sphingomyelin; Cer: Ceramide; SMase: Sphingomyelinase; SMS: Sphingomyelin synthase; Sphk: Sphingosine Kinase; S1P: Sphingosine 1-phosphate; CerK: Ceramide Kinase; C1P: 1-phosphatized ceramide; UGT8: UDP glycosyltransferase 8; GalCer: Galactosylceramide; Lyso-PL: Lysophospholipid; ACSLs: long-chain acyl-CoA synthetase; LPLATs: Lysophospholipid acyltransferase; ACLY: ATP-citrate lyase; ACC: Acetyl-CoA Carboxylase; FASN: Fatty acid synthase; HMGCR: 3-hydroxy-3-methylglutaryl coenzyme a reductase; CH25H: Cholesterol 25-Hydroxylase; 25HC: 25-Hydroxycholesterol; CYP27A1: Cytochrome P450 Family 27 Subfamily A Member 1; 27HC: 27-Hydroxycholesterol.

Lipid metabolism encompasses the synthesis, breakdown, absorption, and modification of lipids to facilitate the processing of substances required for normal physiological functions. The process can be divided into FA metabolism, cholesterol metabolism, PL metabolism, and sphingolipid metabolism (**Figure 1**).

### 1.1 FA metabolism

FAs are a class of biological molecules with long carbon chains, composed of carbon, hydrogen, and carboxyl groups 6. As an energy source, FAs can regulate cell membrane structure and act as signaling molecules. Their synthesis is crucial for the growth and survival of tumor cells by providing essential energy and regulators. Tumor cells mainly obtain endogenous FAs through *de novo* synthesis mediated by FA synthase (FASN) 7. Specifically, mitochondria-derived citrate is first cleaved by ATP citrate lyase to generate cytosolic acetyl-CoA 8. Acetyl-CoA is then carboxylated by acetyl-CoA carboxylase to form malonyl-CoA, which is gradually elongated by FASN to eventually generate long-chain FAs 9. Knockdown of FASN in cancer-associated fibroblasts or treatment with a CD36 antibody can reduce the availability of FAs, thereby inhibiting tumor cell migration. Additionally, cancer-associated fibroblasts promote FA uptake in the tumor microenvironment by inducing the upregulation of FA transport protein 1 (FATP1) in triple-negative breast cancer (TNBC) cells, and FATP1 has been shown to enhance cancer cell proliferation. 10.

Notably, cancer cells exhibit high metabolic plasticity. When de novo FA synthesis is inhibited, they can switch to exogenous uptake pathways to acquire FAs 11. Breast tissue is rich in adipocytes, which can produce and secrete FAs and adipokines, thereby enhancing the invasiveness of cancer cells 12.

### 1.2 Cholesterol metabolism

Cholesterol serves as a critical constituent of biomembranes and lipid rafts, meeting the energy and biosynthetic requirements of rapidly proliferating cancer cells 13. In breast cancer cells, sustained activation of sterol regulatory element-binding protein 2 upregulates genes related to cholesterol biosynthesis. The resulting cholesterol accumulation can suppress CD8^+^ T cell effector functions, thereby weakening cytotoxic immune surveillance and facilitating tumor immune evasion and progression 14. Furthermore, tumor cells maintain a cholesterol-rich tumor microenvironment by modulating cholesterol efflux and uptake pathways 15. Inhibition of proprotein convertase subtilisin/kexin type 9 has been linked to the suppression of tumor growth by lowering serum cholesterol levels, whereas tumor cells enhance cholesterol synthesis and uptake to support rapid proliferation 16.

In addition, cholesterol-derived metabolites can regulate tumor progression. 27-hydroxycholesterol acts as a selective estrogen receptor (ER) modulator, promoting the proliferation and metastasis of ER-positive breast cancer cells 17. Moreover, elevated levels of 25-hydroxycholesterol in tumor-associated macrophages (TAMs) can activate signal transducer and activator of transcription 6-dependent signaling by regulating AMP-activated protein kinase α, thereby promoting the expression of the anti-inflammatory gene arginase 1 18. Therefore, targeting the hydroxylases responsible for producing these metabolites offers a promising novel avenue for immunotherapeutic intervention.

### 1.3 PL metabolism

PLs are fundamental components of biological membranes 19. PL metabolism involves a critical step catalyzed by phospholipase A2, which hydrolyzes PLs to produce lysophospholipids (lyso-PL). In the subsequent reacylation process, long-chain acyl-CoA synthetases (ACSLs) and lysophospholipid acyltransferases (LPLATs) mediate the attachment of a new FA to the sn-2 position of lyso-PL, forming a complete PL 20. Compared to primary tumors, metastatic TNBC tumors contain a greater abundance of unsaturated PLs, particularly phosphatidylcholine and phosphatidylethanolamine (PE) 21. Significantly elevated PE levels in patient plasma have been shown to be positively correlated with poor prognosis in TNBC 22. Furthermore, unsaturated phospholipids can alter membrane fluidity and susceptibility to lipid peroxidation, thereby influencing cellular invasiveness, signal transduction, and sensitivity to ferroptosis 23, 24.

### 1.4 Sphingolipid metabolism

The core molecule of sphingolipid metabolism is ceramide (Cer). On one hand, Cer can inhibit breast cancer progression and enhance chemosensitivity. For example, C6-ceramide markedly promotes docetaxel-induced apoptosis 25. On the other hand, inhibiting excessive Cer accumulation can interfere with tumor metabolism and growth. Yi Xiao et al. found that, compared to normal tissue and non-LAR tumors, LAR-type TNBC tumors exhibit Cer enrichment, with genes involved in ceramide de novo synthesis and degradation being more active. Moreover, under the action of ceramide kinase (CerK) or sphingosine kinase, Cer can be further metabolized into ceramide-1-phosphate (C1P) or sphingosine-1-phosphate (S1P). In invasive cell lines or xenograft models, low levels of CerK significantly inhibit cell migration and slow down tumor growth 26. These findings suggest that CerK and its metabolites play critical roles in the initiation and metastatic progression of primary tumors. For S1P, Xiaozhen Zhang et al. found that inhibiting its levels could reprogram TAMs, attenuate their immunosuppressive phenotype, and increase the population and function of tumor-infiltrating effector T cells, thereby enhancing the synergistic antitumor effects of immune checkpoint blockade 27.

Notably, Cer is galactosylated in the endoplasmic reticulum to form galactosylceramide (GalCer). GalCer is a key sphingolipid, and inducing its degradation can inhibit the development of obesity-related TNBC 28. Cer can also conjugate with oligosaccharide chains to form glycosphingolipids (GSLs) 29. The biosynthesis of GSLs is a glucose-dependent metabolic pathway that is essential for *in vivo* CD8^+^ T cell responses, with their blockade weakening antitumor immunity 30. Reducing GSL levels has been shown to enhance IFN-γ-mediated growth inhibition and proinflammatory signaling, thus effectively augmenting the antitumor proliferative responses and effector functions of natural killer (NK) cells and CD8^+^ T cells 31.

## 2. Lipid Metabolism Modulators and Breast Cancer

### 2.1 FASN and breast cancer

FASN is expressed at higher levels in breast cancer tissues, with a significant increase in expression observed in brain metastases 32. This may reflect the limited availability of exogenous free FAs in the brain, forcing tumor cells that metastasize to the brain to upregulate FASN and enhance endogenous lipid synthesis, thereby meetting demands for membrane biogenesis and metabolic needs 33. FASN-mediated lipid metabolism is regulated by SREBF1. It has been demonstrated by Furuta et al. that AKT activation under hypoxia promotes SREBP1-mediated FASN transactivation, supporting tumor growth in lipid-deprived settings 34. This facilitates tumor survival and expansion in the hypoxic and lipid-restricted environments. FASN is not only associated with advanced cancer and metastasis, but also with the regulation of anti-tumor immunity. Handong Sun et al. reported that FASN expression in cancer cells from male BC (MBC) was significantly higher than in female BC (FBC) samples. In MBC samples, the interaction between cancer cells and T cells was approximately twofold higher than in FBC samples, along with significant activation of TGF-β signaling, suggesting that FASN upregulation may promote an immunosuppressive phenotype by enhancing tumor-immune cell communication 35. Additionly, Zhiwen Qian et al. found that the expression of FASN in breast cancer is positively correlated with the immune-cold tumor microenvironment. Knockdown of FASN can promote GPX4 degradation-induced ferroptosis, thereby enhancing the efficacy of anti-programmed cell death protein 1 immunotherapy 36.

HER2 induces enhanced FA synthesis, thereby maintaining tumor cell growth, proliferation, and metastasis. High expression of FASN in HER2-positive breast cancer is associated with poor progression-free survival. TVB-2640 is the first small-molecule, highly selective FASN inhibitor to enter human clinical trials. It induces tumor cell apoptosis through short interfering RNA and FASN inhibition, restoring drug resistance and inhibiting tumor growth 37. Furthermore, the combination of FASN-targeting drugs and trastuzumab has demonstrated significant antitumor activity in HER2-positive breast cancer models 38.

FASN catalyzes the synthesis of the long-chain saturated FA palmitate, consuming NADPH as a reducing agent. During early malignant transformation, FASN utilizes NADPH in isocitrate dehydrogenase 1 (IDH1)-dependent reductive carboxylation to neutralize reactive oxygen species, thereby promoting cancer cell growth 39. Barbara Schroeder et al. demonstrated that FASN affects cancer cell survival by fine-tuning the mitochondrial apoptotic threshold in a BH3 protein-dependent manner. When FASN is inhibited, disturbances in palmitate/NADPH metabolism induce redox imbalance, which marked reduction in the apoptosis threshold and increased initiation of mitochondrial apoptosis 40. Inhibition of FASN activity can also induce endoplasmic reticulum stress, leading to a significant reduction in ERα levels and altered subcellular localization in endocrine-resistant cells, thereby blocking the growth and cell cycle progression of tamoxifen-resistant breast cancer cells 41. Therefore, FASN is involved in both maintaining metabolic homeostasis and regulating cellular susceptibility to apoptosis, positioning it as a promising candidate target for targeted therapy in breast cancer.

### 2.2 CD36 and breast cancer

CD36 is a FA transporter, and the mature CD36 protein consists of 471 amino acids 42. Overexpression of CD36 promotes FA uptake by enhancing mitochondrial oxidative phosphorylation and lipid synthesis, thereby conferring metabolic plasticity and leading to drug resistance 43. CD36 preferentially mediates the uptake of monounsaturated FAs. Palmitoylated CD36, acting as a lipid saturation sensor, plays a crucial protective role during high-fat diet-accelerated breast cancer metastasis 44. Studies have indicated that co-culture with adipocytes enhances CD36 expression in breast cancer cells, enhance FA uptake, and stimulate FA β-oxidation 45, 46. This observation suggests that stromal cells in the tumor microenvironment may act as important exogenous regulators of tumor cell CD36 expression. Moreover, CD36 plays a role in tumor-infiltrating immune cells. Haiping Wang et al. found that high levels of CD36 enables regulatory T cells to adapt to the tumor microenvironment with high lactate levels, and inhibits the production of pro-inflammatory cytokines 47.

Recently, in HER2-positive breast cancer found that FA uptake is associated with acquired resistance. CD36 upregulation following HER2-targeted therapy (e.g., lapatinib) could serve as a marker of poor prognosis. Mechanistically, resistant cells sustain survival via CD36-dependent FA uptake, inhibiting CD36 to restore lapatinib sensitivity 48, 49. Notably, CD36 suppression specifically targets lapatinib-resistant cells *in vivo* and *in vitro* without impacting sensitive cells 48. Francesca Ligorio et al. also demonstrated in clinical trials that CD36 inhibition can significantly reduce sensitivity to lapatinib 50. This further supports the potential of CD36 as a therapeutic target for overcoming drug resistance. Moreover, in ER-positive breast cancer, the efficacy of tamoxifen is regulated by CD36, and inhibition of CD36 can produce a synergistic effect 51.

Furthermore, CD36-mediated endocytosis is critical for the cellular uptake of challenging therapeutics, such as PROTACs and other large, polar bRo5 molecules 52, 53. Zhengyu Wang et al. enhanced the affinity of drug molecules for CD36 to more efficiently exploit the endocytic pathway and overcome the cell membrane barrier. This led to a 7.7-22.3 fold increase in intracellular drug delivery and a 16.2-23.1 fold improvement in efficacy 54. This strategy of optimizing drug design based on CD36 expression levels in patient tumor tissues holds promise for enabling more precise personalized treatment.

### 2.3 ACSL4 and breast cancer

The lipid metabolism enzyme ACSL4 induces ferroptosis by promoting the esterification of polyunsaturated FAs to acyl-coenzyme A (acyl-CoA) 55. ACSL4 can serve as a biomarker of hormone resistance in human breast cancer 56. In breast cancer cell lines and tumor specimens, ACSL4 mRNA expression has been shown to negatively correlate with ER, androgen receptor (AR), and HER2. In metastatic TNBC cells, the upregulation of ACSL4 drives membrane phospholipid remodeling, which triggers CD47-dependent lipid raft localization and integrin β1 activation. Inhibition of ACSL4 suppresses tumor growth and metastasis and enhances chemosensitivity 21. Collectively, these findings indicate that ACSL4-mediated PL remodeling facilitates metastasis in TNBC, and that inhibiting ACSL4 may be a promising approach to enhance chemotherapy efficacy in TNBC.

However, the role of ACSL4-driven ferroptosis susceptibility in tumor immunity is a double-edged sword. On the one hand, promoting tumor cell ferroptosis can enhance the sensitivity to immunotherapy. IFN-γ released by CD8^+^ T cells can upregulate ACSL4 expression in tumor cells, thereby mediating immunogenic tumor ferroptosis and enhancing the sensitivity to immune checkpoint therapy 57. Therefore, patients with high ACSL4 expression may respond better to chemotherapy due to their increased sensitivity to ferroptosis. Previous studies have demonstrated that among women with a negative family history, the group with high ACSL4 expression was significantly more likely to achieve pathological complete response compared to other groups 58. On the other hand, ferroptosis in cancer cells can produce phospholipid peroxides, which have a potent immunosuppressive effect on dendritic cells, weakening anti-tumor immune responses 59. This suggests that this process may produce opposite immunological consequences in different tumor microenvironments. Therefore, this duality highlights the need for patient stratification and combination treatment strategies. Patients with tumors exhibiting high ACSL4 expression or a pro-immunogenic microenvironment may benefit from therapies that selectively induce ferroptosis, potentially in combination with immune checkpoint inhibitors. Conversely, in other contexts, therapeutic approaches may need to include agents that mitigate these effects, such as tumor-targeted antioxidants or modulators of dendritic cell function.

### 2.4 Others

Aquaporins (AQPs) are membrane proteins responsible for water and glycerol transport. AQP7 deletion leads to significant alterations in pathways, such as lipid metabolism and glutathione metabolism within tumor cells 60. In breast cancer, AQP7 regulates the stress response of cancer cells, and its expression level is associated with overall survival. Furthermore, downregulation of AQP7 expression in a mouse model of breast cancer was shown to suppress primary tumor growth and reduce lung metastasis 61. Separately, the lipid transport protein BLTP2, which facilitates cellular lipid transfer, exhibits a positive correlation between its expression level and the degree of breast cancer malignancy 22.

## 3. Conclusion

Breast cancer development involves critical processes including immune escape, aberrant signaling pathways, and ferroptosis. With advances in small-molecule analytical techniques, lipid metabolism reprogramming has been shown to be closely related to breast cancer. Current studies indicate that multiple lipid metabolism-related proteins, such as CD36, FASN, and ACSL4, show considerable potential as therapeutic targets in breast cancer. Targeting these key enzymes or transporters may yield new clinical interventions. However, the specific mechanisms by which lipid metabolism contributes to breast cancer progression and treatment resistance remain to be fully elucidated. The main challenges include resistance to targeted therapies, difficulties in precise drug delivery due to tumor heterogeneity, and the inherent complexity and compensatory mechanisms of lipid metabolism pathways. With advances in technologies such as lipidomics and spatial metabolomics, a growing number of studies are characterizing the heterogeneity of lipid metabolism in breast cancer at a systems level, along with its relationship to molecular subtypes, the immune microenvironment, and treatment response. These insights offer new possibilities for precise subtyping and outcome prediction in breast cancer. In the future, integrating lipid metabolic profiles with conventional molecular subtypes (such as ER and HER2 status) and with treatment outcomes may help identify patient subgroups that are more sensitive to metabolic-targeted therapies or combination therapy. Systematically elucidate the regulation of lipid metabolism during the occurrence and development of breast cancer, providing key scientific evidence for the formulation of effective and precise treatment strategies.

## Author contributions

Tong-Tong Liu completed the literature review and organization, and drafted the manuscript. Meng-Meng Wang provided suggestions for improvement and offered overall guidance for the article.

## Figures and Tables

**Figure 2:**
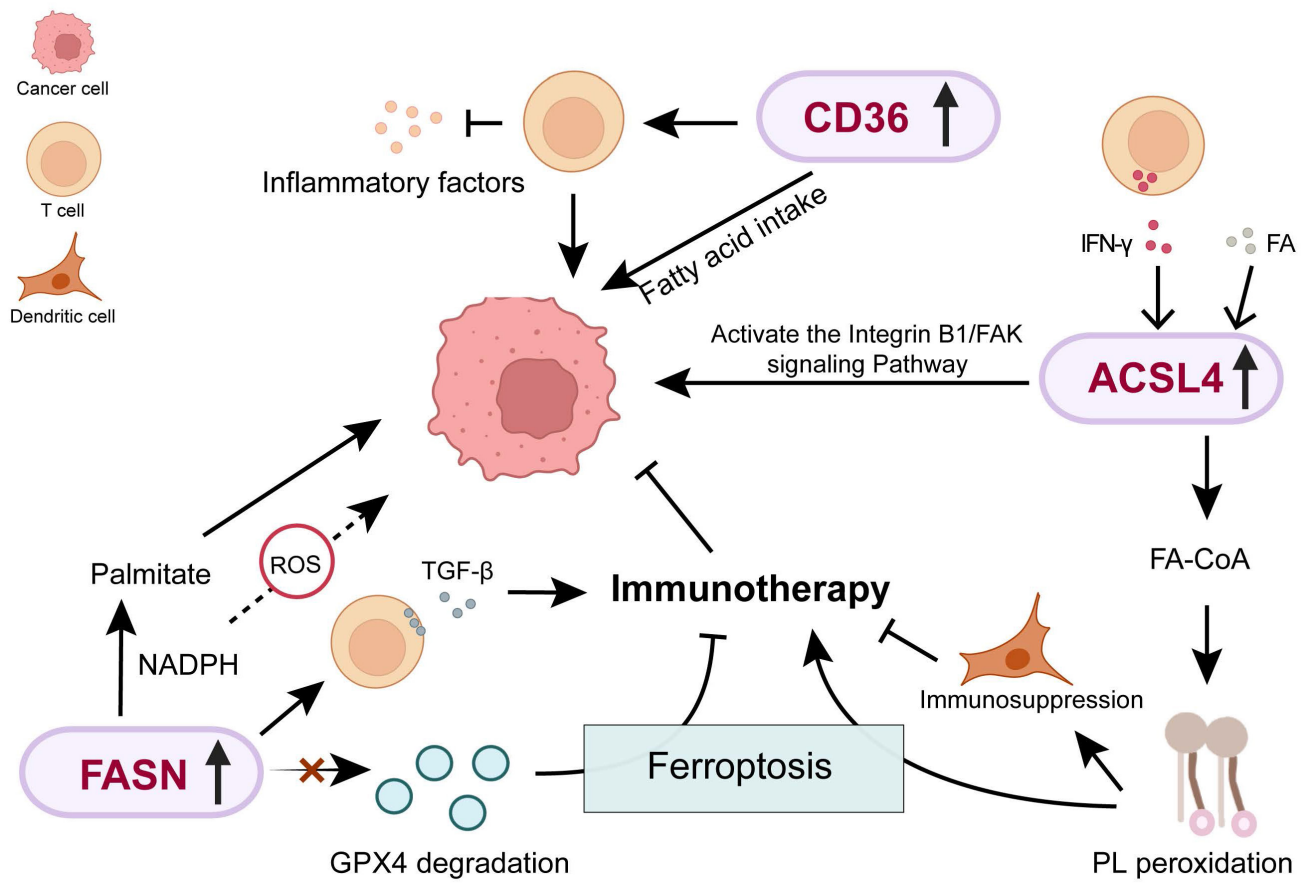
The role of FASN, CD36 and ACSL4-mediated lipid metabolism in breast cancer progression. ROS: Reactive oxygen species; Acetyl-CoA: Acetyl coenzyme A; GPX4: Glutathione peroxidase 4.

## Abbreviations

FASN: fatty acid synthase
CD36: cluster of differentiation 36
ACSL4: acyl-CoA synthetase long-chain family member 4
FA: fatty acids
PL: phospholipids
TNBC: triple-negative breast cancer
Cer: ceramide
lyso-PL: lysophospholipids
NK: natural killer
ACSLs: long-chain acyl-CoA synthetase
LPLATs: lysophospholipid acyltransferase
